# Does Liquid/Injectable Platelet-Rich Fibrin Help in the Arthrocentesis Treatment of Temporomandibular Joint Disorder Compared to Other Infusion Options? A Systematic Review of Randomized Clinical Trials

**DOI:** 10.3390/bioengineering11030247

**Published:** 2024-03-02

**Authors:** Alexander Nemeth, Bruno Vasconcelos Gurgel, Adam Lowenstein, Luiz Juliasse, Rafael S. Siroma, Zoe Zhu, Jamil Awad Shibli, Carlos Fernando Mourão

**Affiliations:** 1 Division of Dental Research Administration, Tufts University School of Dental Medicine, Boston, MA 02111, USA; alexander.nemeth@tufts.edu (A.N.); bruno.grugel@tufts.edu (B.V.G.); adam.lowenstein@tufts.edu (A.L.); 2 Department of Dentistry, Federal University of Rio Grande do Norte, Natal 59056-000, Brazil; luiz.juliasse.053@ufrn.edu.br; 3 Department of Periodontology, Dental Research Division, Guarulhos University, Guarulhos 07023-070, Brazil; rafaelshinoske@gmail.com (R.S.S.); jshibli@ung.br (J.A.S.); 4 Department of Periodontology, Tufts University School of Dental Medicine, Boston, MA 02111, USA; zoe.zhu@tufts.edu

**Keywords:** arthrocentesis, temporomandibular joint disorder, platelet-rich fibrin, i-PRF, liquid PRF

## Abstract

Temporomandibular joint disorders (TMDs) are prevalent musculoskeletal conditions involving pain and dysfunction of jaw mobility and function, which have proven difficult to treat satisfactorily. The present study aimed to assess the effectiveness of a liquid platelet-rich fibrin (i-PRF) infusion during arthrocentesis versus other options using coadjuvant materials to reduce TMD symptoms. A literature search was conducted using PubMed, EMBASE, Web of Science, Scopus, and ClinicalTrials.gov for RCTs published before January 2024, comparing i-PRF to any other TMD treatment. This systematic review was registered on PROSPERO (CRD42023495364). The searches generated several recent RCTs that compared i-PRF injection combined with arthrocentesis (AC) to AC-only or AC with platelet-rich plasma (PRP). The outcomes analyzed included measures of pain (visual analog scale, VAS), maximum mouth opening, joint sounds, and MRI-verified changes in joint structure. Across the RCTs, the addition of i-PRF injection to AC resulted in significant improvements in pain relief, joint function, mouth opening, and structural changes compared to AC-only or with PRP over follow-up periods ranging from 6 to 12 months. Current clinical evidence favors using i-PRF as an adjunct to AC rather than AC-only or AC with PRP for the treatment of TMDs. The improvements in subjective and objective outcome measures are clinically meaningful. Still, additional high-quality RCTs with larger sample sizes and longer follow-ups are required to strengthen the evidence base and better define the role of i-PRF in TMD management guidelines.

## 1. Introduction

The temporomandibular joint (TMJ) is a joint located in front of the ears on both sides of the head. It connects the mandible to the skull and is responsible for a range of movements, such as opening and closing of the mouth, chewing, speaking, and yawning. The TMJ is a complex joint that enables us to perform these daily activities with ease [1].

Temporomandibular joint disorders (TMDs) represent a prevalent and multifactorial group of musculoskeletal and neuromuscular conditions involving the TMJ and/or associated structures with a reported overall prevalence of approximately 31% for adults/elderly and 11% for children/adolescents [2,3]. Symptoms of TMDs include pain, restricted mouth opening (maximal mouth opening (MMO)), joint clicking, and muscular dysfunction, significantly impacting patients’ quality of life [4]. Despite numerous therapeutic approaches ranging from conservative management to surgical interventions, achieving satisfactory outcomes remains challenging due to the complex etiology and varied clinical presentations of TMDs.

The comprehensive treatment strategy employs a multidisciplinary approach, focusing on enhancing jaw mobility, relieving pain, and preventing secondary functional disability and joint damage [5,6]. Initial non-invasive methods, such as habit control, pharmacotherapy, dental treatments, occlusal splints, and physiotherapy, are followed by minimally invasive techniques, including intraarticular treatment (i.e., arthrocentesis), known for their safety and reduced morbidity compared to complex surgeries [7,8]. Arthrocentesis is one of the least invasive and simplest methods for removing inflammatory mediators and reducing pressure inside the joint [9].

Recent advancements in regenerative medicine have inspired interest in blood concentrates such as platelet-rich plasma (PRP) and platelet-rich fibrin (PRF) as potential therapeutic options in orthopedics and dentistry, including TMDs [10,11]. The motivation for using autologous blood products stems from their abundant supply of growth factors and cytokines, such as Platelet-Derived Growth Factor (PDGF), Transforming Growth Factor-β (TGF-β), and Vascular Endothelial Growth Factor (VEGF). These components are crucial in modulating inflammatory responses, enhancing tissue regeneration, and accelerating the healing process through the stimulation of cell proliferation and migration, promotion of angiogenesis, and facilitation of matrix synthesis [12,13]. 

The use of PRF, a second-generation platelet concentrate, is advantageous due to its ease of application and enhanced tissue penetration, especially in its liquid form (liquid PRF or i-PRF). The sustained release of growth factors and cytokines at the injury site not only aids in the quick resolution of inflammatory responses but also enhances the overall regeneration of damaged tissues [13,14]. This highlights the potential of liquid PRF/i-PRF as a biologically potent treatment option for TMDs, paving the way for investigating the clinical efficacy of liquid PRF in TMD management. This approach positions i-PRF as a promising candidate for intraarticular TMJ injections aimed at modulating inflammatory responses and promoting tissue healing. However, the clinical effectiveness of liquid PRF in managing TMDs still requires further exploration [7,15]. To date, few clinical studies have assessed the effects of the treatment outcomes of intraarticular injection of injectable platelet-rich fibrin (i-PRF) after arthrocentesis [15,16,17]. 

The present study aimed to assess the effectiveness of the liquid PRF infusion during arthrocentesis versus other options using coadjuvant materials to reduce TMD symptoms. In this research, the authors evaluated changes in patient-related outcomes, such as pain intensity, MMO, joint clicking, and other functional parameters following liquid PRF therapy, based on randomized clinical trials (RCTs).

## 2. Materials and Methods

The protocol for this review was conducted following the Preferred Reporting Items for Systematic Reviews and Meta-Analyses (PRISMA) protocols and was registered with PROSPERO under the registration number CRD42023495364.

### 2.1. Focused Question

In patients with TMDs, is the application of liquid PRF during arthrocentesis procedures more effective than the infusion of other materials in the TMJ area for reducing symptoms of TMJ disorders?

### 2.2. Eligibility Criteria

Criteria for inclusion were determined using the PICOS format (Patients, Intervention, Comparison, Outcome, and Study design) and are as follows:

**P**: Patients with TMD;
**I**: Application of liquid PRF/i-PRF in the area of the TMJ;
**C**: Any other treatment (e.g., hyaluronic acid (HA));
**O**: Reduction of symptoms of temporomandibular disorder (e.g., noise, maximal mouth opening, pain);
**S**: Randomized clinical trials.

There were no restrictions regarding the date of publication. Criteria for excluding articles were as follows: (1) Studies that did not directly compare i-PRF to another treatment (i.e., arthrocentesis); (2) Studies that were not randomized clinical trials; and (3) non-English language studies.

### 2.3. Search Strategy

An electronic search for relevant articles published before 1 January 2024, was completed using the PubMed/MEDLINE, EMBASE, Web of Science, and Scopus databases. Grey literature was searched using clinicaltrials.gov. Additionally, the reference list of included studies was examined for potentially relevant studies. The search terms used were as follows: ((i-PRF) OR (injectable PRF) OR (iPRF) OR (liquid PRF) OR (l-PRF) OR (lPRF)) AND ((temporomandibular disorder) OR (TMJ disorder)). A comprehensive list of the utilized search strategies for each database is shown in Table 1.

### 2.4. Study Selection and Data Extraction

First, studies were screened through the analysis of titles and abstracts. Then, studies that met the eligibility criteria were selected to be read in full to verify they met the eligibility criteria. This was completed by one researcher (A.N.) and checked by a second researcher (C.M.).

Data extraction was conducted by A.N. and reviewed by C.M. Any disagreements were resolved through careful discussion. Data extracted included authors (with year of publication), patient conditions, treatment groups, number of subjects (with sex, age), comparator group, follow-up, interventions, outcomes measured, and main results reported by the authors that related to TM symptoms. Any missing information was attempted to be obtained by contacting the study authors. 

### 2.5. Risk of Bias

The study evaluated potential biases by examining the randomization process, the adherence to interventions as intended, any missing outcome data, how outcomes were measured, and the selection of reported results. 

The quality of the chosen RCTs was evaluated to determine the potential for bias. The risk of bias within studies was assessed independently by two reviewers (A.N. and C.M.) using the Cochrane risk-of-bias tool for randomized trials (RoB 2) across the following domains: (1) bias arising from randomization; (2) bias due to deviations from intended interventions; (3) bias due to missing outcome data; (4) bias in the measurement of the outcome; and (5) bias in the selection of the reported result. Within each domain, studies were rated as having “low”, “some concerns”, or “high” risk of bias. The overall bias judgment was determined based on the interpretations of bias in individual domains [18]. Any discrepancies were settled through a guided discussion among the authors. 

## 3. Results

A qualitative analysis and synthesis of outcomes were carried out. Due to heterogeneity in the study methods and results, along with the limited number of relevant randomized clinical trials, it was not possible to conduct a meta-analysis of the data.

### 3.1. Study Selection

**Figure 1:**
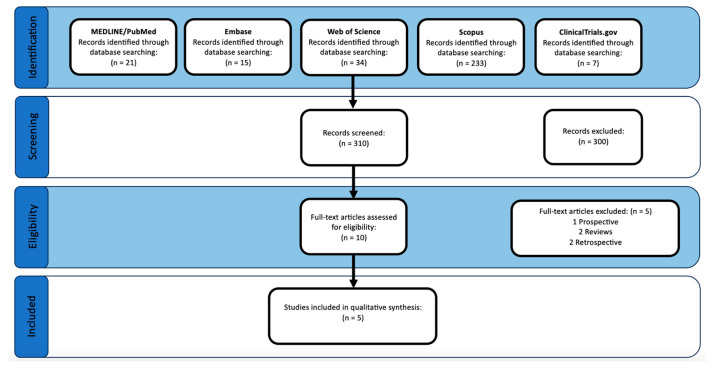
PRISMA study flow diagram.

The initial search produced a total of 310 studies (21 from MEDLINE/PubMed, 15 from Embase, 31 from Web of Science, 233 from Scopus, and 7 from ClinicalTrials.gov). After initial screening, 10 articles were selected for full-text analysis. Following full-text analysis, five articles were excluded for study design reasons (one prospective (no control group), two reviews, two retrospectives). Thus, five studies were selected to be included in this qualitative review [15,16,17,19,20]. The search flow can be seen in Figure 1.

### 3.2. Inter-Rater Agreement on Risk of Bias

The risk of bias within studies was assessed using Cohen’s kappa statistic to evaluate inter-rater agreement on risk ratings. The two independent raters almost entirely agreed with each other, with a kappa value of 0.89. This high level of agreement indicates a very low risk of bias in the ratings of study quality and risk of bias across the included randomized controlled trials.

### 3.3. Study Characteristics

The characteristics of the five included studies are summarized in Table 2. Based on the inclusion and exclusion criteria, all the studies included were RCTs. Although there was no limit on the date of publication, all the studies included were published between 2021 and 2023. The studies used treatments of AC followed by one to several i-PRF injections that ranged from one injection immediately following AC to i-PRF injected at one-month intervals for six and/or twelve months. Across the studies, the i-PRF group was directly compared to either AC-only or AC followed by PRP injections mimicking the i-PRF injections. In total, between the five studies, a total of 202 individuals with TMDs were studied: 150 females, 38 males, and 14 unspecified genders. The average age of treatment groups in the studies varied from 26.45 to 47.2 years old. The four studies that reported on the gender of participants contained both male and female participants, with a single study not reporting gender. The sample sizes of the treatment groups ranged from 7 to 38 individuals. The length of the follow-up periods ranged from 3 months to 12 months. The outcomes measured in the studies included pain (VAS, Visual Analogic Scale), movement (MMO, Maximum Incisal Opening (MIO), ipsilateral, contralateral, protrusive), presence or absence of sounds, HCDS (Helkimo Clinical Dysfunction Score), and MRI (Magnetic Resonance Imaging) evaluation of disc position and joint effusion. Only one study reported attrition, losing 6 participants due to an incomplete follow-up [15,16,17,19,20].

### 3.4. Treatment Procedures

The procedures across the studies were somewhat varied. In the four studies with an AC-only treatment group, the AC-only group received a single treatment of AC. In two of these studies, the AC plus i-PRF group received an initial AC treatment and Magnetic Resonance Imaging immediately followed by an i-PRF injection, then i-PRF injections on a weekly basis for 3 additional weeks [15,17]. In the other two studies, the AC/i-PRF combination group received AC immediately, followed by a single i-PRF injection [16,19]. One study compared AC in combination with PRP or i-PRF. In this study, the groups received AC followed immediately by PRP or i-PRF, then an injection of the respective treatment at 1-month intervals for a total of 6 months of treatment [20].

### 3.5. Arthrocentesis (AC) Procedures

The AC procedures also varied slightly across the studies. Two of the studies that had an AC-only group followed the procedure according to Nitzan and Dolwick (1991) [21] where they used a two, 20-gauge needle system in which they placed the first needle 10 mm in front of the tragus and 2 mm below the canthus–tragus line. Then, 2–3 mL of saline solution was injected through the first needle. The second needle was inserted 20 mm in front of the tragus, and 6 mm below the canthus–tragus line, and a total of 200 mL of saline solution was injected into the superior space of the TMJ [15,17], flowing in via the first needle and out via the second needle. The other two other studies with an AC-only group followed a similar but slightly different procedure. They used a two, 20-gauge needle system where they placed the first needle 10 mm in front of the tragus and 2 mm below the canthus–tragus line. Then, 2 mL of lactated Ringer solution was injected through the first needle. The second needle was then inserted 20 mm in front of the tragus and 6 mm below the canthus–tragus line, and 100 mL of 5% lactation solution was injected into the superior space of the TMJ, flowing in via the first needle and out via the second needle [16,19]. The single study that only used AC in combination with PRP or i-PRF used a system similar to that of Nitzan and Dolwick (1991) [21], where they used a two-needle system with a 200 mL Ringer lactate solution [20].

### 3.6. Blood Concentrate Procedures

The i-PRF preparations were nearly identical across the studies, with only slight differences in technique. All the studies produced the i-PRF by centrifuging blood at 700 rpm for 3 min and removing the top layer (i-PRF) [15,16,17,19,20]. In all the studies with an AC-only group, the i-PRF was injected following the AC procedure through the more posterior needle after removal of the anterior needle [15,16,17,19,20]. They varied slightly in the amount of i-PRF injected. Two studies with an AC-only group injected 1 mL of i-PRF into the superior joint compartment [15,17]. One study had an AC-only group injected at a maximum of 2 mL [19]. The final study with an AC-only group injected 1.5 mL of i-PRF [16]. The single study comparing AC with i-PRF to AC with PRP removed blood and prepared the i-PRF in the same method as other studies. However, instead of injecting the i-PRF using a needle from the AC procedure, they used a 26-gauge needle to inject 2 mL of i-PRF into the superior joint space 10 mm anterior and 2 mm posterior to the tragus (same location as other studies). The PRP was prepared with 10 mL of blood mixed with 1 mL of 3.8% sodium citrate solution and centrifuged at 1000 rpm for 7 min. The upper layer, including the buffy coat, was then transferred to a new tube and centrifuged at 3000 rpm for 10 min. Following the second spin, lower one-third (PRP) was collected, and 2 mL of the PRP was injected into the superior joint space using a 26-gauge needle 10 mm anterior and 2 mm posterior to the tragus (same as the i-PRF group) [20]. 

### 3.7. Pain 

All five studies examined pain via VAS. In the four studies comparing AC-only to AC in combination with i-PRF, the combination AC plus i-PRF group showed statistically significantly greater decreases in pain as measured by VAS at every time point measured from as short as 7 days postoperatively to as long as 12 months postoperatively [15,16,17,19]. The single study comparing AC in combination with PRP or i-PRF found a statistically significant difference in pain reduction found at 1 week after the 2nd and 3rd injections (months 1 and 2 after starting treatment) between the PRP and i-PRF groups with the PRP group having a greater decrease in pain. During the follow-up appointments after the 3rd to 6th injections and at the 9th month, there were no significant differences in pain between the groups [20]. 

A single study analyzed the pain using the Helkimo Clinical Dysfunction Score (HCDS). This study compared AC-only to AC in combination with i-PRF and found a statistically significantly greater decrease in HCDS at all postoperative time points (10 days, 30 days, and 3 months postoperative) in the AC plus i-PRF group compared to the AC-only group [19].

### 3.8. Movement

All five studies measured movement in some way. In the four studies comparing AC-only to AC in combination with i-PRF, the combination of AC plus i-PRF group generally showed that i-PRF was more effective in increasing all movements. Two studies showed statistically significant greater increases in MMO, contralateral movements, ipsilateral movements, and protrusive movements postoperatively compared to AC only at 1st, 2nd, 3rd,6th, and 12th months postoperatively [15,17]. One study found statistically significant greater increases in MIO, RLM (Right Lateral Movement), and LLM (Left Lateral Movement) at 1 week, 3 months, and 6 months postoperatively in the i-PRF plus AC group compared to the AC-only group [16]. The study comparing i-PRF plus AC to AC-only found a statistically significant difference in the increase in MIO at 30 days postoperative. 

The combination treatment had a greater increase in MIO at that time point. It is worth noting that the differences at the other time points measured (10 days and 3 months postoperatively) were close to significant (*p* = 0.077 and *p* = 0.081, respectively), with the i-PRF group showing a greater MIO increase [19]. The single study comparing AC in combination with PRP or i-PRF found no statistically significant differences were found between groups at the 1st and 2nd follow-ups (months 1 and 2). However, a statistically significant difference between MMOs was found from the 3rd follow-up onward, with the i-PRF in combination with the AC group having a greater increase in MMO than the PRP in combination with the AC group. Additionally, this study found no statistically significant differences between groups for lateral or protrusive movement improvement [20].

### 3.9. Sounds and/or Clicking

Two studies looked at sounds/clicking of the TMJ, one comparing AC-only to AC in combination with i-PRF and one comparing AC in combination with PRP or i-PRF. The study comparing AC-only to AC plus i-PRF found the combination treatment had a statistically significantly greater improvement in clicking symptoms at all time points measured (1 week, 3 months, and 6 months postoperatively) [16]. The study comparing AC in combination with i-PRF or PRP found no statistically significant difference between the two groups in the improvement of clicking sounds [20].

### 3.10. Disc Position and Joint Effusion

A single study conducted an MRI evaluation looking at disc position and joint effusion, comparing AC in combination with PRP or i-PRF. This study found “marked changes toward normal disc position” in 1 patient in the PRP + AC group and 5 patients in the i-PRF + PRP group [20]. Additionally, no significant differences in joint effusion were found between groups. However, both groups had decreased joint effusion [20].

### 3.11. Risk of Bias within Studies

**Figure 2:**
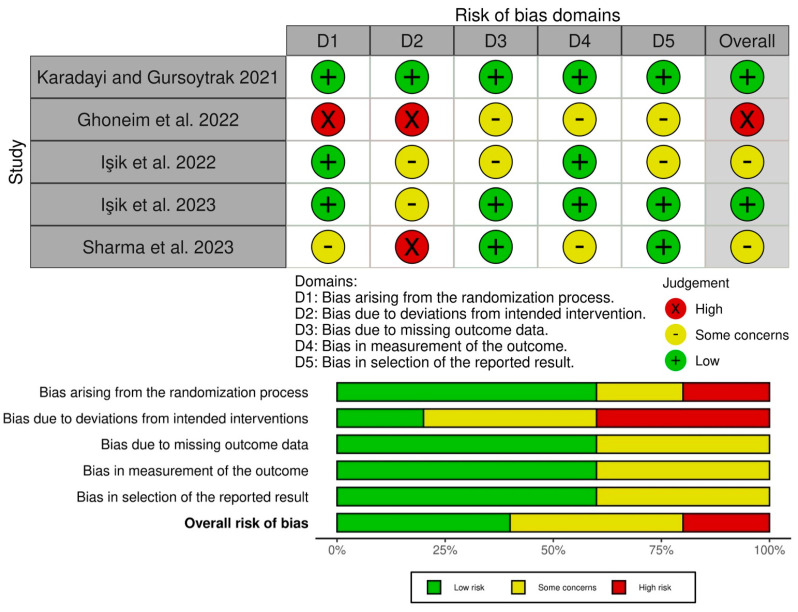
Risk of bias of the studies based on Cochrane’s RoB2 [15,16,17,19,20].

The risk of bias within studies, as based on Cochrane’s RoB2, is shown in Figure 2. Two studies included were determined to have a low overall risk of bias [17,19], two were determined to have some concerns [15,20], and one had a high risk of bias [16]. Across studies, the most common domain with a high to medium (some concerns) risk of bias was bias due to deviations from the intended intervention (D2). Overall, the bias in the selection of reported results, bias in the measurement of outcome, bias due to missing outcome data, and bias arising from the randomization process were favorable for each individual study.

## 4. Discussion

The management of TMDs represents a significant challenge within the realm of oral and maxillofacial surgery, requiring interventions that not only aim to alleviate the immediate symptoms experienced by patients but also strive to restore the overall functionality of the jaw [22,23]. This comprehensive systematic review specifically targets the evaluation of injectable platelet-rich fibrin (i-PRF) as a supplementary treatment to arthrocentesis (AC), with the goal of enhancing treatment efficacy in reducing TMD symptoms. These symptoms encompass pain, limited mouth opening, and joint clicking, which significantly impact patients’ quality of life. Arthrocentesis, characterized as a minimally invasive procedure to lavage the joint space, is acknowledged for its effectiveness in relieving pain and improving joint mobility [16,24]. However, the lingering symptoms post-treatment signal the necessity for adjunctive therapies that can extend the benefits of the initial procedure. The blood concentrates, distinguished by their rich concentration of growth factors, are hypothesized to significantly amplify the healing outcomes of AC by stimulating tissue regeneration and mitigating inflammation more effectively than traditional treatment modalities [25,26]. This research selected randomized clinical trials for examination [15,16,17,19,20], ensuring a thorough evaluation of liquid PRF/i-PRF’s contribution to TMD management, thereby reflecting a dedication to grounding advancements in treatment within the framework of evidence-based practice. The analysis of recent studies suggests that adding i-PRF to TMD treatment protocols could result in more effective management strategies for this debilitating condition.

The management of TMJ disorders heavily relies on the concept of viscosupplementation, particularly when applied to the superior or inferior compartments of the joint. It is vital to choose the appropriate area for injection, and the superior joint space is often preferred due to its accessibility and the extent to which it is affected by the disorder. By reinstating the joint’s natural lubrication mechanism, which is commonly compromised in TMDs, viscosupplementation plays a crucial role in reducing friction and enhancing joint functionality. This approach is essential for alleviating various symptoms, including pain, restricted movement, and joint clicking, underscoring its importance in the comprehensive management of TMJ disorders.

New methods in viscosupplementation involve using blood concentrates like i-PRF instead of traditional viscosupplementation materials, such as sodium hyaluronic acid [27,28]. Injectable platelet-rich fibrin is a modern alternative that not only provides lubrication but also contains growth factors that help promote tissue regeneration and facilitate healing. This dual functionality makes i-PRF a highly attractive option for treating TMJ disorders, addressing both symptomatic and etiological aspects of these conditions [7,15,16,17,19,20]. Furthermore, i-PRF is derived from the patient’s own blood [29], enhancing its significance and advancing treatment methodologies within the field of TMJ disorder management.

The findings of this systematic review underscore the efficacy of viscosupplementation, particularly through the use of i-PRF in conjunction with arthrocentesis for the treatment of temporomandibular joint disorders. The treatment protocols highlighted across the included studies emphasize the strategic integration of i-PRF injections following AC, aiming to capitalize on the regenerative potential of i-PRF to bolster the outcomes of standard AC procedures. This combined approach is substantiated by significant enhancements in patient-related outcomes, including reductions in pain, increases in maximum mouth opening, and decreases in joint clicking. The 2 to 6+ point reduction in pain scores on 10-point VAS scales indicates significant changes in discomfort that can significantly enhance a patient’s quality of life [15,16,17,19,20]. Similarly, an increase of seven millimeters or more in the interincisal distance can improve the ease and range of jaw motion during activities such as chewing and speaking [15,17]. When these changes are sustained over 6–12 months, along with decreased joint noise and easier lateral movements, as seen in this case, they can lead to considerable gains in day-to-day activities and improve the overall quality of life. While there may be variations in treatment protocols, the evidence suggests that using i-PRF alongside arthrocentesis can meaningfully improve patient-centered outcomes, and it should be integrated into TMD management guidelines. Further trials can be conducted to optimize treatment protocols. These improvements align with the broader literature, which supports the synergistic benefits of combining mechanical lavage with biological agents to foster healing and functional recuperation of the TMJ.

The deployment of i-PRF as a form of viscosupplementation marks a significant shift from traditional substances like hyaluronic acid, providing not only mechanical support and lubrication but also a suite of growth factors that facilitate tissue regeneration. This dual-action mechanism is congruent with the existing literature findings, suggesting that the therapeutic advantages of i-PRF emanate from its capacity to modulate the inflammatory milieu within the TMJ and promote the body’s inherent healing processes [30,31]. Furthermore, the observed procedural variability across studies, from the timing and frequency of i-PRF injections to the nuances of the AC technique, mirrors a customized treatment approach that accommodates the unique requirements of TMD patients [15,16,17,19,20]. This adaptability underscores the potential for personalized medicine in the nuanced management of this complex disorder.

The use of i-PRF in treating TMJ disorders harnesses a rich spectrum of growth factors and cytokines critical for tissue repair and regeneration, such as PDGF, TGF-β, and VEGF. Those growth factors are instrumental in this regenerative cascade. PDGF stimulates cellular proliferation in fibroblasts and smooth muscle cells, while TGF-β regulates collagen and proteoglycan synthesis to facilitate cartilage formation. VEGF promotes angiogenesis to improve blood supply and nutrition to joint tissues [13,14,32]. Together, these elements initiate tissue repair by reducing inflammation, encouraging cartilage regeneration, and improving vascularity. Compared to traditional viscosupplements like HA or PRP, i-PRF provides a more sustained release of these bioactive growth factors due to its intrinsic fibrin architecture, enabling durable joint healing [33]. The infusion of growth factors such as PDGF, TGF-β, and VEGF into the TMJ through i-PRF injections can trigger a regenerative process that encourages cell growth and angiogenesis and modulates the inflammatory response. This holistic approach not only provides symptomatic relief but also underpins the TMJ’s inherent repair mechanisms, delivering a thorough and efficacious treatment strategy [20]. In particular, the application of i-PRF in the articular disc targets fibroblasts and other cellular elements, promoting the regeneration of disc tissue, reducing inflammation, and facilitating tissue healing. This concerted action is pivotal for re-establishing the disc’s structural integrity and functionality, ensuring the smooth operation of the TMJ and thus mitigating prevalent symptoms of TMD, such as pain and movement restriction. Through these mechanisms, i-PRF emerges as a cornerstone in the treatment of TMJ disorders, offering a promising avenue for achieving long-term relief and functional recovery.

The advantages of i-PRF over traditional viscosupplements raise the intriguing possibility of exploring intraarticular i-PRF injection as a single-modality treatment for temporomandibular disorders without an obligatory arthrocentesis precursor. A significant limitation of the available evidence is the lack of randomized controlled trials examining intraarticular i-PRF injection alone, without AC. While early findings highlight the benefits of complementing arthrocentesis with i-PRF administration, none of the studies evaluated has assessed i-PRF injections in isolation [15,16,17,19,20]. Thus, the solitary impacts of i-PRF on symptomology and joint function remain undefined. This constrains determinations of whether initial arthrocentesis is a requisite precursor for i-PRF therapy. However, robust clinical evidence to support or refute this approach is currently lacking. While early findings highlight the benefits of complementing joint rinsing with i-PRF administration, no randomized controlled trials have examined i-PRF injections in isolation. The results of this review suggest the potential for a beneficial impact of solitary i-PRF administration on joint function and symptomology akin to that achieved through combined protocols. Still, high-quality prospective studies and trials with adequate long-term follow-up are imperative to investigate the viability of stand-alone i-PRF injection for TMD management. Confirming the efficacy of this streamlined intervention could cement i-PRF’s role as a frontline biologic for TMJ disorders.

In the present study, it was possible to gain an overview of i-PRF’s use as a coadjuvant material after arthrocentesis treatment. In summary, i-PRF offers key advantages over other viscosupplementation options that substantiate its growing usage alongside arthrocentesis for comprehensive TMD treatment. However, there are some limitations to this analysis. The main limitations of the available evidence are the small number of RCTs and the lack of any RCT study groups receiving i-PRF without prior arthrocentesis. Additional limitations include the overall small sample sizes, short follow-up times, potential for publication bias, and heterogeneity in the study protocols, which constrain the strength of the conclusions. Variability in arthrocentesis methods, i-PRF centrifugation and injection parameters, and outcome measure reporting across trials introduce possible confounders. Nevertheless, the totality of current evidence favors an adjuvant role for i-PRF in AC-based treatment protocols for temporomandibular joint disorders. Further well-designed clinical trials delineating precise regenerative protocols may cement the role of i-PRF as a frontline biologic therapy for TMJ disorders.

This systematic review has some limitations worth noting. The analysis was constrained due to the small number of randomized controlled trials available. However, the authors comprehensively synthesized the existing evidence base. The studies included in our review generally had modest sample sizes and limited follow-up durations, which restricted the strength of conclusions about the long-term impacts on the temporomandibular joint. Furthermore, there was some heterogeneity in the study protocols regarding the specifics of the arthrocentesis methods, i-PRF preparation, injection timing/frequency, and outcome assessments. While qualitative synthesis helped account for this, standardized treatment and evaluation protocols in future studies could facilitate quantitative meta-analysis. Finally, there may have been publication bias favoring positive treatment effects, and only studies published in English were included, which restricted geographic representativeness and potentially excluded relevant non-English research. Despite these limitations, the results consistently supported an adjuvant role for i-PRF alongside arthrocentesis over arthrocentesis alone. Further randomized trials are needed to address these limitations and define optimal protocols more conclusively.

## 5. Conclusions

This systematic review provides evidence supporting the integration of i-PRF with arthrocentesis for the treatment of temporomandibular joint disorders. The study suggests that using i-PRF in addition to arthrocentesis results in better outcomes across several measures over a period of 6–12 months.

The review shows that adding i-PRF injections to standard arthrocentesis protocols leads to significant improvements in pain relief, mouth opening, joint function, and favorable structural changes compared to arthrocentesis alone or combined with other agents like PRP. These differences align with the idea that i-PRF helps to reduce inflammation and promote healing through targeted growth factor delivery.

While the results support the use of i-PRF in TMJ treatment, more research is needed. Few studies have investigated using i-PRF alone without prior lavage. Further trials with longer follow-up periods are necessary to determine the long-term impact of i-PRF on joint homeostasis. Nevertheless, the study provides evidence that using i-PRF may help alleviate symptoms and restore jaw function in TMD patients who have not responded to other treatments.

## Figures and Tables

**Table 1:** Search strategies for each database.

Database	Search Strategy
PubMed/MEDLINE	(((“Platelet-Rich Fibrin”[Mesh]) OR (((injectable[All Fields] OR “injectable”[All Fields] OR i[All Fields] OR liquid[All Fields] OR l[All Fields]) AND (“Platelet-Rich Fibrin”[Mesh] OR PRF[All Fields]))))) AND (((((“Temporomandibular Joint Disorders”[Mesh])) OR (temporomandibular[All Fields] disorder[All Fields])) OR (TMJ[All Fields] disorder[All Fields])))
EMBASE	(‘platelet rich fibrin’/exp OR (((injectable OR ‘injectable’ OR i OR liquid OR l) AND (‘platelet rich fibrin’ OR prf)))) AND ((‘temporomandibular joint dysfunction’/exp) OR (temporomandibular AND disorder) OR (tmj AND disorder))
Web of Science	TS=((injectable OR “injectable” OR i OR liquid OR l) AND (“platelet-rich fibrin” OR prf)) AND TS=(((“temporomandibular joint disorder *”) OR (temporomandibular AND disorder *) OR (tmj AND disorder *)))
Scopus	((TITLE-ABS-KEY(injectable OR “injectable” OR i OR liquid OR l) AND TITLE-ABS-KEY(“platelet-rich fibrin” OR prf)) AND (TITLE-ABS-KEY((“temporomandibular joint disorder *”) OR (temporomandibular AND disorder *) OR (tmj AND disorder *))))
ClinicalTrials.gov	(injectable OR “injectable” OR i OR liquid OR l) | (Platelet-Rich Fibrin OR PRF) AND (temporomandibular OR tmj) AND (disorder OR dysfunction)

**Table 2:** Included studies in the systematic review.

Study (Year)	Patient Conditions	Treatment Groups	Number of Subjects (F, M, Mean Age)	Follow-Up (d = days, m = Months)	Interventions (i-PRF Treatment vs. Comparator Treatments)	Outcomes Measured	Main Results
Karadayim, Gursoytrak (2021) [19]	Internal derangement of the TMJ	i-PRF + AC vs. AC-only	E (i-PRF + AC): 18 (10 F, 8 M, 39.97). AC-only (18 9 F, 9 M, 39.67)	Preoperative, 10 d, 30 d, 3 m	AC followed immediately by 1 intraarticular injection of i-PRF. 1 treatment of AC.	Pain: VAS HCDS Movement: MIO	VAS (E mean: 5.83 ± 2.550, C mean: 2.94 ± 2.043, *p* < 0.001), HCDS (E mean: 1.361 ± 5.158, C mean: 9.22 ± 6.916, *p*: 0.039)
Ghoneim, et al. (2022) [16]	TMJ joint displacement	i-PRF + AC vs. AC-only	i-PRF + AC: 20 (16 F, 4 M, 26.45) AC-only 20 (13 F, 7 M, 28.60)	Preoperative, 7 d, 3 m, 6 m	AC followed immediately by 1 intraarticular injection of i-PRF. 1 treatment of AC.	Pain: VAS Sounds: presence or absence Movement: MIO, RLM, LLM	Significant reduction in pain, increase in MIO, and improvement in lateral movements in i-PRF group compared to AC group (*p* < 0.05)
Isik, et al. (2022) [15]	TMJ joint osteoarthritis	i-PRF + AC vs. AC-only	i-PRF + AC: 18 (16 F, 2 M, 44.67) AC-only (18, 17 F, 1 M, 45.72)	Preoperative, 1 m, 2 m, 3 m, 6 m, 12 m	AC plus 4 consecutive intraarticular injections of i-PRF on a weekly basis (AC-only performed with the first injection). 1 treatment of AC.	Pain, MMO, jaw movements	Significant differences in pain, MMO, and jaw movements over 12 months (*p* < 0.001) in the group with i-PRF injections
Isik, et al. (2023) [17]	TMJ disc displacement	AC + i-PRF vs. AC-only	AC + i-PRF: 38 (34 F, 4 M, 47.2) AC-only (38 35 F, 3 M, 46.8)	Preoperative, 1 m, 2 m, 3 m, 6 m, 12 m	AC plus 4 consecutive intraarticular injections of i-PRF on weekly basis (AC only performed with first injection). 1 treatment of AC.	Pain reduction, jaw movement	Treatment success: AC 73.7%, AC + i-PRF 100%, Pain and jaw movement: Significant improvement in AC + i-PRF (*p* < 0.001)
Sharma, et al. (2023) [20]	Internal derangement of the TMJ	AC + PRP: vs. AC + i-PRF	AC + PRP (7, Age: 20–50) AC + i-PRF: 7 (Age: 20–50)	Preoperative, 1 week after treatment for 6 m of treatment, 9 m	AC followed by i-PRF at 1-month intervals for 6 months. AC followed by PRP at 1-month intervals for 6 months.	Pain: VAS Movement: MMO, Lateral, Protrusive Sounds: presence or absence MRI Evaluation: Disc position JE	VAS, MMO, lateral movement, protrusive movement: Statistically significant improvements in both groups, more in i-PRF group (*p* < 0.05)

Legend: **TMJ:** Temporomandibular joint; **i-PRF**: Injectable platelet-rich fibrin; **AC:** Arthrocentesis; **E**: Experimental group; **C**: Control group; **VAS**: Visual Analog Scale; **HCDS**: Helkimo Clinical Dysfunction Score; **MIO:** Maximum Incisal Opening; **MMO**: Maximum mouth opening; **JE**: Joint effusion; **PRP**: Platelet-rich plasma; **RLM:** Right Lateral Movement; **LLM:** Left Lateral Movement; **m:** months.

## Data Availability

Data are available from the corresponding author upon request.
